# Различия в экспрессии микроРНК в плазме крови у пациентов с генетически подтвержденным синдромом множественных эндокринных неоплазий 1 типа и их фенокопиями

**DOI:** 10.14341/probl13357

**Published:** 2024-01-24

**Authors:** Д. А. Трухина, Е. О. Мамедова, А. Г. Никитин, Ф. А. Кошкин, Ж. Е. Белая, Г. А. Мельниченко

**Affiliations:** Национальный медицинский исследовательский центр эндокринологии; Национальный медицинский исследовательский центр эндокринологии; Научно-исследовательский институт пульмонологии; Медико-генетический центр «Геномед»; Национальный медицинский исследовательский центр эндокринологии; Национальный медицинский исследовательский центр эндокринологии

**Keywords:** микроРНК, синдром множественных эндокринных неоплазий 1 типа, фенокопии, аденома гипофиза, первичный гиперпаратиреоз

## Abstract

**ОБОСНОВАНИЕ:**

ОБОСНОВАНИЕ. Синдром множественных эндокринных неоплазий 1 типа (МЭН-1) — редкое аутосомно-доминантное заболевание, обусловленное мутациями в гене MEN1, кодирующем белок менин. Этот синдром характеризуется возникновением опухолей околощитовидных желез, гастроэнтеропанкреатических нейроэндокринных опухолей, аденом гипофиза, а также других эндокринных и неэндокринных опухолей. Если пациент с МЭН-1 фенотипом не имеет мутаций в гене MEN1, то такое состояние рассматривается как фенокопия синдрома (фМЭН-1). Возможной причиной этих изменений могут быть изменения в эпигенетической регуляции, в частности в экспрессии микроРНК, которые могут влиять на сигнальные пути менина.

**ЦЕЛЬ:**

ЦЕЛЬ. Определить циркулирующие микроРНК, различно экспрессирующиеся в плазме крови у пациентов с генетически подтвержденным синдромом МЭН-1, его фенокопиями и контролем.

**МАТЕРИАЛЫ И МЕТОДЫ:**

МАТЕРИАЛЫ И МЕТОДЫ. Выполнено одноцентровое исследование типа «случай-контроль». Проведена оценка экспрессии микроРНК в плазме крови пациентов с генетически подтвержденным синдромом МЭН-1 (гМЭН-1), фМЭН-1 и относительно здорового контроля. Забор крови проводился натощак, образцы цельной крови однократно центрифугировались и хранились при температуре -80°С. Выделение тотальной РНК проводили с помощью набора miRNeasy Mini Kit с QIAcube. Библиотеки готовили с помощью набора QIAseq miRNA Library Kit в соответствии с инструкцией производителя. Секвенирование циркулирующих микроРНК проводили на Illumina NextSeq 500 (Illumina). Последующую обработку данных проводили с помощью биоинформационного алгоритма DESeq2.

**РЕЗУЛЬТАТЫ:**

РЕЗУЛЬТАТЫ. Всего в исследование были включены в группу гМЭН-1 — 21 пациент, в группу фМЭН-1 — 11 пациентов, в группу контроля — 12 относительно здоровых добровольцев. Медиана возраста в группе гМЭН-1 составила 38,0 лет [34,0; 41,0]; в группе фМЭН-1 — 59,0 [51,0; 60,0]; в группе контроля — 59,5 [51,5; 62,5]. Все группы не отличались по полу (р=0,739) и индексу массы тела (р=0,116). Группа гМЭН-1 отличалась по возрасту от пациентов фМЭН-1 и контрольной группы (p<0,001). В результате высокопроизводительного секвенирования было получено 25 различно экспрессирующихся микроРНК в группах гМЭН-1 и фМЭН-1 (21 микроРНК с повышенной экспрессией, 4 — с пониженной). При сравнении образцов от групп фМЭН-1 и относительно здорового контроля выявлено 10 различно экспрессирующихся микроРНК: 5 — с повышенной экспрессией в группе фМЭН-1 по сравнению с контрольной группой, 5 — с пониженной. В группах гМЭН-1 и контроля обнаружено 26 различно экспрессирующихся микроРНК: 24 с повышенной экспрессией в группе гМЭН-1 по сравнению с контролем, 2 — с пониженной. Для дальнейшей валидации результатов методом RT-qPCR были отобраны наиболее отличающиеся по экспрессии микроРНК среди групп (в группах гМЭН-1 и фМЭН-1 — miR-3613-5p, miR-335-5p, miR-32-5p, miR-425-3p, miR-25-5p, miR-576-5p, miR-215-5p, miR-30a-3p, miR-141-3p, miR-760, miR-501-3p; в группах гМЭН-1 и контроля — miR-1976, miR-144-5p miR-532-3p, miR-375; а также в группах фМЭН-1 и контроля — miR-944, miR-191-5p, miR-98-5p).

**ЗАКЛЮЧЕНИЕ:**

ЗАКЛЮЧЕНИЕ. В ходе пилотного исследования методом высокопроизводительного секвенирования обнаружены микроРНК, экспрессия которых может отличаться у пациентов с гМЭН-1, фМЭН-1 и контролем. Полученные результаты нуждаются в валидации при помощи другого метода оценки экспрессии микроРНК на большей выборке пациентов.

## Abstract (en)

**BACKGROUND:**

BACKGROUND: MEN-1 is a rare autosomal dominant disease caused by mutations in MEN1 gene encoding the menin protein. This syndrome is characterized by the occurrence of parathyroid tumors, gastroenteropancreatic neuroendocrine tumors, pituitary adenomas, as well as other endocrine and non-endocrine tumors. If a patient with the MEN-1 phenotype carry no mutations in the MEN1 gene, the condition considers a phenocopy of syndrome (phMEN1). The possible cause of this changes could be changes in epigenetic regulation, particularly in microRNA expression that might affect menin signaling pathways.

**AIM:**

AIM: to identify differently expressed circulating miRNAs in plasma in patients with genetically confirmed MEN-1 syndrome, its phenocopies and healthy controls.

**MATERIALS AND METHODS:**

MATERIALS AND METHODS: single-center, case-control study was conducted. We assessed plasma microRNA expression in patients with genetically confirmed MEN-1 (gMEN1), phMEN1 and healthy controls. Morning plasma samples were collected from fasting patients and stored at –80°C. Total RNA isolation was performed using miRNeasy Mini Kit with QIAcube. The libraries were prepared by the QIAseq miRNA Library Kit following the manufacturer. Circulating miRNA sequencing was done on Illumina NextSeq 500 (Illumina). Subsequent data processing was performed using the DESeq2 bioinformatics algorithm.

**RESULTS:**

RESULTS: we enrolled 21 consecutive patients with gMEN1 and 11 patients with phMEN1, along with 12 gender matched controls. Median age of gMEN1 was 38,0 [34,0; 41,0]; in phMEN1 — 59,0 [51,0; 60,0]; control — 59,5 [51,5; 62,5]. The gMEN1 group differed in age (p<0.01) but not gender (р=0.739) or BMI (р=0.116) compared to phMEN1 and controls group, the last two groups did not differ by these parameters (p>0.05). 25 microRNA were differently expressed in groups gMEN1 and phMEN1 (21 upregulated microRNAs, 4 — downregulated). Comparison of samples from the phMEN-1 group and relatively healthy controls revealed 10 differently expressed microRNAs: 5 — upregulated; 5 — downregulated. In the gMEN-1 and control groups, 26 differently expressed microRNAs were found: 24 — upregulated; 2 — downregulated. The miRNAs most differing in expression among the groups were selected for further validation by RT-qPCR (in the groups of gMEN1 vs phMEN1 — miR-3613-5p, miR-335-5p, miR-32-5p, miR-425-3p, miR-25-5p, miR-576-5p, miR-215-5p, miR-30a-3p, miR-141-3p, miR-760, miR-501-3p; gMEN1 vs control — miR-1976, miR-144-5p miR-532-3p, miR-375; as well as in phMEN1 vs control — miR-944, miR-191-5p, miR-98-5p).

**CONCLUSION:**

CONCLUSION: In a pilot study, we detected microRNAs that may be expressed differently between patients with gMEN-1 and phMEN-1. The results need to be validated using different measurement method with larger sample size.

## ОБОСНОВАНИЕ

Синдром множественных эндокринных неоплазий 1 типа (МЭН-1) — аутосомно-доминантное заболевание, вызванное мутацией в гене-супрессоре MEN1, находящемся на длинном плече хромосомы 11q13, который кодирует белок менин. Распространенность синдрома МЭН-1, по некоторым данным, составляет 3–20 на 100 тыс. чел. с одинаковым распределением между мужским и женским полом [1]. Заболевание характеризуется сочетанием опухолей околощитовидных желез (ОЩЖ), нейроэндокринных опухолей (НЭО) желудочно-кишечного тракта (чаще всего поджелудочной железы), гипофиза, а также другими эндокринными и неэндокринными опухолевыми образованиями [2]. Встречающиеся герминальные мутации в гене MEN1 как в семейных (около 90%), так и в спорадических случаях (около 10%) синдрома МЭН-1 рассеяны по всей кодирующей области и сайтам сплайсинга, корреляции между генотипом и фенотипом отсутствуют. Проявления заболевания возникают в разные моменты времени в разных органах у людей, несущих идентичные мутации, что может объясняться теорией «двойного удара» Кнудсона, а также влиянием других факторов, вызывающих индивидуальное развитие онкогенеза [3–6]. Однако от 5% до 10% пациентов с МЭН-1-подобными проявлениями не имеют мутаций в кодирующей области MEN1, что может быть связано с крупными делециями данного гена, мутациями в промоторе и прочих нетранслируемых областях гена MEN1 [3][7][8]. Если у пациента с клиническими проявлениями МЭН-1 не удалось обнаружить мутацию в гене MEN1, то данное состояние считается фенокопией синдрома МЭН-1. Причина сочетания нескольких эндокринных МЭН-1-ассоциированных опухолей у таких пациентов остается не до конца изученной, и как возможное основание возникновения такого фенотипа могут быть рассмотрены мутации в таких генах, как CDKN1A, CDKN1B, CDKN2B, CDKN2C, CDC73, CASR, RET, AIP, других еще неизвестных генах, а также эпигенетические изменения или случайное сочетание нескольких опухолей у одного пациента [1][2][6][9].

Эпигенетические модификации представляют собой область большого интереса, поскольку они играют роль в сверхэкспрессии онкогенов или подавлении генов-супрессоров, в результате чего происходит стимуляция сигнальных путей, ответственных за образование опухолей [10][11]. Известно, что гиперметилирование сайтов CpG в промоторных областях, модификация гистонов генов-супрессоров опухолей может приводить к потере функции этих генов [12–15]. Сайленсинг генов, опосредованный некодирующими РНК (нкРНК), является еще одним возможным эпигенетическим механизмом образования опухолей, в том числе МЭН-1-ассоциированных, поскольку нкРНК играют роль во многих биологических процессах, например в транскрипции, сплайсинге, трансляции, экспрессии генов, клеточном цикле, импринтинге, эмбриогенезе [16].

МикроРНК являются наиболее подробно изученным классом нкРНК. Они представляют собой короткие нкРНК и регулируют экспрессию генов, обычно связываясь с определенными участками в 3’ нетранслируемых областях (3’-НТО) мРНК, а также в 5’-НТО, кодирующей области или промоторе [17], с различным уровнем комплементарности, и либо вызывая деградацию, либо ­блокируя трансляцию мРНК [18][17]. Многие микроРНК присутствуют в жидкостях организма (включая сыворотку, плазму, мочу и др.) и играют роль в межклеточной коммуникации, функционируя как гормоноподобные молекулы, влияя на поведение различных клеток [19][20].

Мутации в гене MEN1 у пациентов с синдромом МЭН-1 могут быть ассоциированы с включением целого ряда эпигенетических изменений, в том числе компенсаторного характера, учитывая не 100% пенетрантность развития симптомокомплекса, а также различное фенотипическое представление у пациентов с одной и той же мутацией. Кроме того, у пациентов с фенокопиями синдрома МЭН-1, возможно, реализуется ухудшение экспрессии гена MEN1, в том числе на эпигенетическом уровне [21].

Таким образом, целью нашего пилотного исследования было выявить различно экспрессирующиеся циркулирующие микроРНК в плазме крови у пациентов с генетически подтвержденным синдромом МЭН-1, его фенокопиями и здоровым контролем.

## МАТЕРИАЛЫ И МЕТОДЫ

## Место и время проведения исследования

Исследование проведено на базе отделений нейроэндокринологии и остеопороза и остеопатий ГНЦ РФ ФГБУ «НМИЦ эндокринологии» Минздрава России. Высокопроизводительное секвенирование микроРНК, а также их выделение выполнено на базе лаборатории «Геномед». Биоинформатический анализ полученных данных выполнен на базе ФГБУ «НИИ пульмонологии» ФМБА России. Были использованы образцы, полученные с апреля 2019 г. по январь 2022 г.

## Изучаемые популяции

В исследование были включены пациенты с генетически подтвержденным синдромом МЭН-1 (гМЭН-1) и пациенты с фенокопиями синдрома МЭН-1 (фМЭН-1), а также относительно здоровый контроль (К). Предварительного расчета выборки не проводилось. Пациенты были разделены на 3 группы: набор в первые две группы определялся результатом генетического исследования, 3-я группа представляла собой здоровых добровольцев без клинических проявлений эндокринных заболеваний.

## Способ формирования выборки: сплошной.

В случае выявления у пациента фенотипа МЭН-1 при отсутствии мутаций в гене MEN1, а также других генах, ответственных за проявления такого фенотипа, состояние расценивалось как фенокопия данного синдрома. Наиболее часто при фенокопиях МЭН-1 имеется сочетание аденомы гипофиза (АГ) и опухоли околощитовидной железы. Таким образом, в группу фМЭН-1 были включены: пациенты обоих полов, от 18 лет и старше, в активной стадии (без лечения, либо с отсутствием ремиссии на фоне лечения) акромегалии и первичного гиперпаратиреоза (ПГПТ) (наиболее частое сочетание опухолей); с отсутствием мутаций по данным метода высокопроизводительного секвенирования (NGS) в панели генов-кандидатов (MEN1, CDKN1A, CDKN1B, CDKN1C, CDKN2A, CDKN2C, CDKN2D, AIP, SDHA, SDHB, SDHC, SDHD, PRKAR1A, GNAS, PRKCA, POU1F1, CASR, CDC73); отсутствием крупных делеций/дупликаций кодирующей области MEN1 по данным мультиплексной амплификации лигированных зондов (MLPA).

Критерии исключения в группе фМЭН-1: возраст моложе 18 лет, лучевая терапия в анамнезе, ремиссия на фоне приема препаратов, беременность, тяжелые системные заболевания.

В группу гМЭН-1 включались пациенты с генетически подтвержденным синдромом МЭН-1 методом секвенирования по Сэнгеру или методом NGS, подходящие по полу к группе фМЭН-1, и обязательно с наличием сочетания АГ+ПГПТ как проявление синдрома.

В качестве контрольной группы выбраны относительно здоровые добровольцы, подходящие по полу и возрасту к группе фМЭН-1.

## Дизайн исследования

Проведено одноцентровое одномоментное выборочное исследование.

## Описание медицинского вмешательства

Пациентам проводился забор цельной крови утром натощак в пробирку с EDTA K2. В течение 30 мин после забора крови образцы цельной крови однократно центрифугировались (лабораторная центрифуга Eppendorf 5810R с комплектом роторов (А-4-81, Ф-4-81-MTP/Flex, FA-45-30-11 и F-45-48-PCR)) при температуре +5 °С на скорости вращения 3000 об/мин в течение 20 мин. Далее образцы плазмы раскапывались в криопробирки, замораживались и хранились при температуре -80 °C.

Выделение микроРНК из плазмы крови проводили с помощью miRNeasy Serum/Plasma Kit (Qiagen, Германия) согласно инструкции компании-производителя на автоматической станции QIAcube (Qiagen, Германия). Для предотвращения деградации в выделенную РНК добавляли 1 ед. RiboLock RNase Inhibitor (Thermo Fisher Scientifiс, США) на 1 мкл раствора нуклеиновых кислот. Концентрацию суммарной РНК в водном растворе оценивали на спектрофотометре NanoVue Plus (GE Healthcare, Великобритания). Для дальнейшей работы отбирали образцы с концентрацией суммарной РНК в водном растворе не ниже 5 нг/мкл. Экспрессию микроРНК анализировали с помощью секвенирования на Illumina NextSeq 500 (Illumina NextSeq 500, США). Библиотеки были подготовлены с помощью QIAseq miRNA Library Kit в соответствии со стандартными протоколами производителя. Контроль качества библиотек выполнялся на Lab Chip GX. Биоинформационная обработка была следующей: адаптеры удалялись с помощью Cutadapt; полученные файлы FASTQ были затем картированы на геном человека (сборка GRCh37) с помощью bowtie2. FastQC использовался в качестве инструмента для визуализации различных измерений контроля качества. Для каждого образца последовательности аннотировались с использованием баз данных человеческих пре-микроРНК и зрелых микроРНК, предоставленных в miRBase (http://microrna.sanger.ac.uk/sequences/), с помощью SeqBuster. TargetScan, Diana-TarBase v8 и mirPath v.3, были использованы для предсказания мишеней. Платформа miRNet 2.0 использовалась для анализа взаимодействия микроРНК и их генов-мишеней.

## Статистический анализ

Для статистической обработки материала использовались программы Statistica 13.3 (StatSoft США), IBM SPSS 23. Данные описательной статистики представлены в виде медианы, а также 25-го и 75-го перцентилей. Для описания качественных данных рассчитывали абсолютные (n) и относительные значения (%). Нормальность распределения проверялась критерием Шапиро–Уилка. Связь между количественными показателями устанавливали, используя непараметрический метод Краскела–­Уоллиса ANOVA, с поправкой на множественные сравнения Бонферрони (р<0,017). Для анализа связей между категориальными переменными использовали критерий χ-квадрат Пирсона и точный критерий Фишера. Статистически значимыми считали различия при p<0,05. Биоинформатический анализ данных секвенирования выполнен при помощи пакета DESeq2. По достоверности обнаруженных различий микроРНК были разделены на 2 группы: больше 10 прочтений и менее 10 прочтений.

## Этическая экспертиза

Протокол исследования одобрен на заседании локального этического комитета ФГБУ «НМИЦ эндокринологии» Минздрава России от 10 марта 2021 г. (­протокол № 4).

## РЕЗУЛЬТАТЫ

## Объекты (участники) исследования

Всего в исследование были включены 44 человека: в группу гМЭН-1 — 21 пациент, в группу фМЭН-1 — 11 пациентов, в группу К — 12 относительно здоровых добровольцев. Все группы не отличались по полу (р=0,739) и индексу массы тела (ИМТ) (р=0,116). Группа гМЭН-1 отличалась по возрасту от пациентов фМЭН-1 и К-групп (p<0,001) (табл. 1.). Учитывая наличие различий по возрасту между группами, в дальнейшем на полученных результатах применялась поправка на возраст для нивелирования этого фактора.

**Table:** Таблица 1. Характеристика исследуемых групп Примечание: Me [Q1; Q3] — медиана, первый и третий квартили; n — количество пациентов, ИМТ — индекс массы тела.

Параметр	гМЭН-1(n=21)	фМЭН-1 (n=11)	Контроль (n=12)	p-value
Возраст на момент взятия крови, лет, Me [Q1; Q3]	38,0 [ 34,0; 41,0]	59,0 [ 51,0; 60,0]	59,5 [ 51,5; 62,5]	0,0003 p1–3<0,001 p1–2<0,001 p2–3=1,0
Пол, жен, n (%)	17 (80,95)	10 (90,9)	11 (91,7)	0,739
ИМТ, кг/м², Me [Q1; Q3]	24,7 [ 22,1; 30]	28,95 [ 26,7; 35,7]	26,75 [ 23,5; 31,5]	0,116

В группе фМЭН-1 у всех пациентов имелись акромегалия (инсулиноподобный фактор роста 1 560 нг/мл [ 414,3; 679]) и ПГПТ (паратиреоидный гормон 85,86 нг/мл [ 67,41; 140,1]; кальций общий 2,71 ммоль/л [ 2,59; 2,77]; кальций скорр. по альбумину 2,65 ммоль/л [ 2,54; 2,68]) в активной стадии заболеваний. У 3/11 пациентов, кроме поражения гипофиза и ОЩЖ, были выявлены другие эндокринные и неэндокринные образования (табл. 2). Во всех случаях акромегалия была диагностирована раньше, чем ПГПТ; медиана до диагностики второй эндокринной опухоли равна 1 году [ 0; 2].

**Table:** Таблица 2. Характеристика пациентов с множественными образованиями (3/11) в группе фенокопий синдрома множественных эндокринных неоплазий 1 типа Примечание: ГНО — гормонально-неактивное образование; ОЩЖ — околощитовидная железа; СТГ-АГ — соматотропинома, ж — женщины.

№	Возраст, пол	Образования
1	60, ж	СТГ-АГ, аденома правой верхней ОЩЖ, диффузная гиперплазия обоих надпочечников, светлоклеточный рак правой почки 1 ст. (T1bN0M0G2), кисты в левой почке (I, II, IIF, III, IV типы по Bosniak)
2	59, ж	СТГ-АГ, ПГПТ без визуализации, ГНО головки поджелудочной железы, образование правого надпочечника, узелковая гиперплазия левого надпочечника, нейрофиброма забрюшинного пространства справа
3	56, ж	СТГ-АГ, аденомы левой и правой нижних ОЩЖ, интратиреоидная аденома ОЩЖ, ГНО левого надпочечника, гемангиома правой теменной кости

В группе гМЭН-1 у 21 пациента имелись АГ и ПГПТ. По секреции АГ были разделены следующим образом: 10 — пролактиномы (ПРЛ), 4 — гормонально-неактивные АГ (НАГ), 3 — болезнь Иценко–Кушинга (БИК), 2 — смешанные соматотропиномы/пролактиномы (СТГ+ПРЛ), 1 — соматотропинома, 1 — смешанная секреция адренокортикотропного гормона (АКТГ)+ПРЛ. Только АГ и ПГПТ на момент включения в исследование были выявлены у 3/21 пациентов; АГ, ПГПТ, образования поджелудочной железы — у 6/21 пациентов; у остальных пациентов компоненты синдрома МЭН-1 представлены большим количеством эндокринных и неэндокринных поражений и более подробно отображены в таблице 3.

**Table:** Таблица 3. Характеристика пациентов с множественными образованиями в группе генетически подтвержденного синдрома множественных эндокринных неоплазий 1 типа Примечание: ПРЛ-АГ — пролактинома; АКТГ-АГ — кортикотропинома; НАГ — гормонально-неактивная аденома гипофиза; СТГ-АГ — соматотропинома; ПГПТ — первичный гиперпаратиреоз; НЭО — нейроэндокринное образование; ГНО — гормонально-неактивное образование; ОЩЖ — околощитовидная железа; mts — метастазы; ж — женщины; м — мужчины.

№	Возраст, пол	Образования
1	19, м	ПРЛ-АГ, гиперплазия 4 ОЩЖ, множественные НЭО поджелудочной железы, НЭО левого легкого (S6)
2	39, ж	ПРЛ-АГ, гиперплазия 4 ОЩЖ, проинсулинома, ГНО поджелудочной железы, узелковая гиперплазия обоих надпочечников, ГНО латеральной ножки левого надпочечника
3	43, ж	ПРЛ-АГ, гиперплазия 4 ОЩЖ, ГНО хвоста, перешейка, крючковидного отростка поджелудочной железы, нисходящего отдела двенадцатиперстной кишки, НЭО правого легкого (S3–4), ГНО левого надпочечника
4	36, ж	АКТГ/ПРЛ-АГ, аденомы 4 ОЩЖ, аденома ОЩЖ в средостении, множественные гастриномы поджелудочной железы, ГНО левого надпочечника, гигантоклеточная опухоль альвеолярного отростка нижней челюсти, гигантское внеорганное объемное образование брюшной полости и малого таза неясного генеза
5	53, ж	НАГ, аденомы 4 ОЩЖ, ГНО поджелудочной железы (pT3N1Mх, G2), НЭО правого легкого (S1), ГНО правого надпочечника, гемангиома печени (S7)
6	49, ж	АКТГ-АГ, аденомы 3 ОЩЖ, 3 аденомы ОЩЖ в средостении, множественные ГНО поджелудочной железы, НЭО правого легкого (S8/S9), множественные образования печени вторичного характера (mts)
7	39, ж	СТГ/ПРЛ-АГ, гиперплазия 4 ОЩЖ, ГНО тела и хвоста поджелудочной железы, гиперплазия левого надпочечника, образование молочной железы
8	40, м	НАГ, аденомы 4 ОЩЖ, множественные ГНО головки и тела поджелудочной железы, инсулиномы хвоста поджелудочной железы, ГНО левого надпочечника, множественные аденомы левой околоушной железы
9	36, ж	ПРЛ-АГ, аденомы 2 ОЩЖ, ГНО поджелудочной железы, ГНО обоих надпочечников
10	37, ж	ПРЛ-АГ, аденома ОЩЖ, инсулиномы поджелудочной железы (рТ2 (m), рN0 (0/19), сМ1), множественные ГНО поджелудочной железы, ГНО обоих надпочечников
11	41, ж	СТГ/ПРЛ-АГ, аденомы 4 ОЩЖ, инсулиномы поджелудочной железы (G1/G2), ГНО крючковидного отростка поджелудочной железы, ГНО левого надпочечника, папиллярный рак щитовидной железы
12	36, м	ПРЛ-АГ, аденомы 3 ОЩЖ, НЭО правого легкого (S6) G1

## Основные результаты исследования

По результатам биоинформатического и статистического анализов между группами гМЭН-1 и фМЭН-1 были обнаружены 25 различно экспрессирующихся микроРНК: 4 микроРНК были со сниженной экспрессией в группе фМЭН-1 по сравнению с гМЭН-1, а 21 микроРНК — с повышенной, с уровнем значимости p<0,05 после поправки на множественность сравнений. По достоверности обнаруженных различий микроРНК были разделены на группы с более чем 10 прочтений и менее чем 10 прочтений в одной из групп (табл. 4, 5). Тепловая карта экспрессии 25 микроРНК в группах пациентов фМЭН-1 по сравнению с гМЭН-1 представлена на рисунке 1, где видно достаточно четкое распределение по группам большинства ­пациентов.

**Table:** Таблица 4. МикроРНК, различно экспрессирующиеся в плазме крови пациентов с генетически подтвержденным синдромом МЭН-1 и его фенокопиями (более 10 прочтений) Примечание: значения «изменения экспрессии» со знаком «-» означают пониженную экспрессию.

МикроРНК	Изменение экспрессии	p-value	padj
hsa-miR-3613-5p	6,009964	2,77358E-17	>0,0001
hsa-miR-335-5p	5,643685	2,39335E-12	>0,0001
hsa-miR-32-5p	5,405104	1,78086E-13	>0,0001
hsa-miR-760	5,277836	2,66768E-07	>0,0001
hsa-miR-2276-3p	-28,44718	4,95287E-14	>0,0001
hsa-miR-454-3p	-2,926644	0,005807373	0,05

**Table:** Таблица 5. МикроРНК, различно экспрессирующиеся в плазме крови пациентов с генетически подтвержденным синдромом МЭН-1 и его фенокопиями (менее 10 прочтений в одной из групп) Примечание: значения «изменения экспрессии» со знаком «-» означают пониженную экспрессию.

МикроРНК	Изменение экспрессии	p-value	padj
hsa-miR-425-3p	4,729436	5,20358E-09	>0,0001
hsa-miR-25-5p	4,748414	4,21213E-07	>0,0001
hsa-miR-501-3p	4,215495	9,61385E-07	>0,0001
hsa-miR-576-5p	4,472254	1,81309E-06	>0,0001
hsa-miR-1224-5p	4,988417	2,49849E-06	>0,0001
hsa-miR-141-3p	3,582906	1,10466E-05	0,0001
hsa-miR-129-5p	15,62514	4,09791E-05	0,001
hsa-miR-532-3p	3,864718	4,78686E-05	0,001
hsa-miR-3187-3p	4,403906	6,03505E-05	0,001
hsa-miR-503-5p	3,991885	6,95132E-05	0,001
hsa-miR-30a-3p	3,735631	0,00016462	0,002
hsa-miR-4306	3,610371	0,00017717	0,002
hsa-miR-1908-5p	4,373386	0,000448854	0,004
hsa-miR-664a-5p	-3,937607	0,000437559	0,004
hsa-miR-130b-3p	4,091780173	0,001093891	0,01
hsa-let-7d-3p	-3,543134153	0,003363402	0,03
hsa-miR-19a-3p	3,813056169	0,005167953	0,05
hsa-miR-345-5p	3,677793403	0,005579495	0,05
hsa-miR-215-5p	3,451745926	0,007573295	0,05

**Figure:**
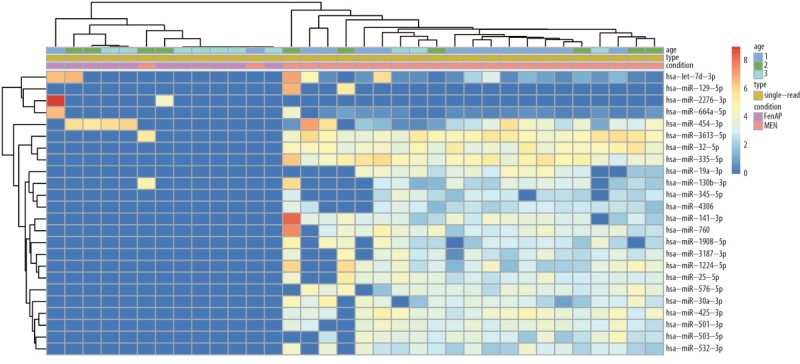
Рисунок 1. Тепловая карта 25 различно экспрессирующихся микроРНК между группами генетически подтвержденного синдрома МЭН-1 (гМЭН-1) и его фенокопий (фМЭН-1). Синий цвет соответствует низкому уровню экспрессии, красный — высокому.

При сравнении образцов от групп фМЭН-1 и относительно здорового контроля выявлено 10 ­различно ­экспрессирующихся микроРНК: 5 — с повышенной экспрессией в группе фМЭН-1 по сравнению с контрольной группой, 5 — с пониженной (табл. 6); количество прочтений всех микроРНК было более 10. В группах гМЭН-1 и контроля обнаружено 26 различно экспрессирующихся микроРНК: 24 — с повышенной экспрессией в группе гМЭН-1 по сравнению с контролем, 2 — с пониженной (табл. 7); в 23 микроРНК количество прочтений в одной из групп было менее 10.

**Table:** Таблица 6. МикроРНК, различно экспрессирующиеся в плазме крови пациентов с фенокопиями синдрома МЭН-1 и контролем Примечание: значения «изменения экспрессии» со знаком «-» означают пониженную экспрессию.

МикроРНК	Изменение экспрессии	p-value	padj
hsa-miR-944	4,56177	0,005307	0,03
hsa-miR-191-5p	1,443402	1,26E-05	0,0007
hsa-miR-151a-3p	1,123178	0,000437	0,01
hsa-miR-103a-3p	0,900005	0,005122	0,04
hsa-miR-486-5p	0,63895	0,008858	0,05
hsa-miR-142-5p	-0,6539	0,007434	0,04
hsa-let-7d-5p	-0,7748	0,005593	0,04
hsa-let-7c-5p	-0,8186	0,005073	0,04
hsa-miR-98-5p	-1,07757	0,003226	0,04
hsa-miR-122-5p	-1,44606	0,001241	0,02

**Table:** Таблица 7. МикроРНК, различно экспрессирующиеся в плазме крови пациентов с генетически подтвержденным синдромом МЭН-1 и контролем Примечание: значения «изменения экспрессии» со знаком «-» означают пониженную экспрессию.

МикроРНК	Изменение экспрессии	p-value	padj
hsa-miR-144-5p	6,085829	8,38E-19	>0,0001
hsa-miR-25-5p	5,200256	1,16E-09	>0,0001
hsa-miR-1976	5,191892	7,2E-09	>0,0001
hsa-miR-576-5p	5,16823	5,49E-10	>0,0001
hsa-miR-30a-3p	5,039024	1,59E-08	>0,0001
hsa-miR-130b-3p	4,965452	5,33E-06	0,0001
hsa-miR-205-5p	4,91427	0,001422	0,02
hsa-miR-3187-3p	4,83344	5,1E-07	>0,0001
hsa-miR-4433a-3p	4,780702	1,3E-05	0,00027
hsa-miR-574-3p	4,728751	5,33E-07	>0,0001
hsa-miR-532-3p	4,665485	4,2E-08	>0,0001
hsa-miR-215-5p	4,294858	9,21E-05	0,002
hsa-miR-1301-3p	4,280196	0,00025	0,004
hsa-miR-598-3p	4,163633	0,000139	0,002
hsa-miR-4306	4,162105	2,24E-06	>0,0001
hsa-miR-326	4,152838	0,002441	0,03
hsa-miR-500a-3p	4,114861	0,001869	0,02
hsa-miR-99a-5p	4,084514	0,001267	0,02
hsa-miR-625-5p	4,074149	0,000349	0,005
hsa-miR-485-5p	4,053361	0,003423	0,03
hsa-miR-345-5p	4,045615	0,000398	0,005
hsa-miR-99b-5p	4,006249	0,000451	0,006
hsa-miR-190b	3,881351	0,003464	0,03
hsa-miR-210-3p	3,787025	0,003668	0,03
hsa-miR-10a-5p	-2,29968	0,005126	0,05
hsa-miR-375	-2,38632	0,000164	0,003

Для дальнейшей верификации полученных результатов на большей группе пациентов методом количественной полимеразной цепной реакции с обратной транскрипцией (RT-qPCR) выбраны одни из наиболее отличающихся по профилю экспрессии микроРНК в группах гМЭН-1 против фМЭН-1: miR-3613-5p, miR-335-5p, miR-32-5p, miR-425-3p, miR-25-5p, miR-576-5p, miR-215-5p, miR-30a-3p, miR-141-3p, miR-760, miR-501-3p; гМЭН-1 против контроля: miR-1976, miR-144-5p miR-532-3p, miR-375; фМЭН-1 против контроля: miR-944, miR-191-5p, miR-98-5p.

Проведенный с помощью miRNet 2.0 [22] анализ показал, что обнаруженные микроРНК, влияя на их гены-мишени, регулируют транскрипционные факторы, молекулы сигнальных путей, клеточные циклы, дифференцировку клеток, канцерогенез и гены-­онкосупрессоры (рис. 2). Большая часть обнаруженных микроРНК связана с процессами онкогенеза и клеточными ­циклами.

**Figure:**
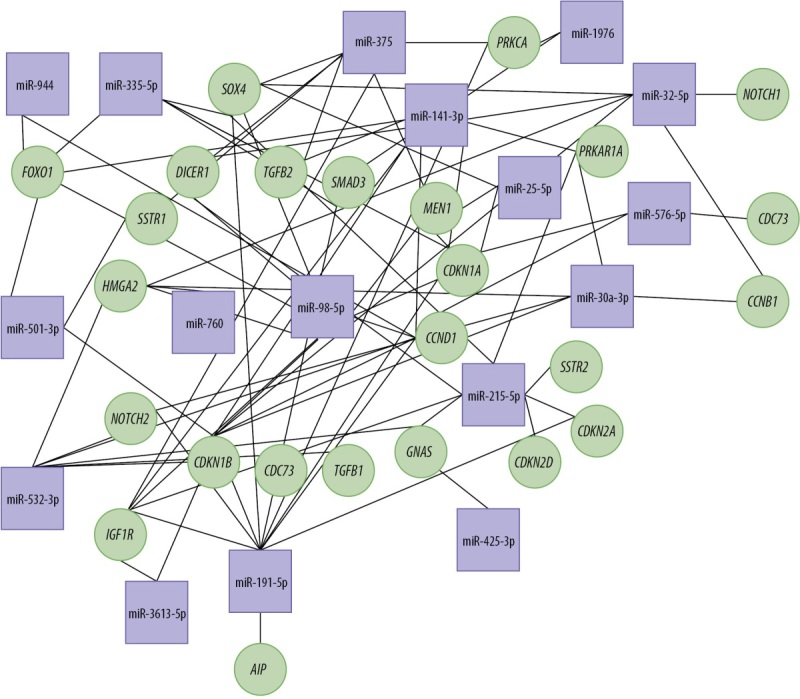
Рисунок 2. Сеть взаимодействия микроРНК и генов-мишеней. В квадратах указаны микроРНК, в кругах — гены-мишени.

## ОБСУЖДЕНИЕ

В нашем исследовании впервые изучены различия в экспрессии циркулирующих микроРНК плазмы крови у пациентов с гМЭН-1 и фМЭН-1 методом высокопроизводительного секвенирования, а также проведено их сравнение с группой контроля. Получено более 20 различно экспрессирующихся микроРНК при сравнении групп гМЭН-1 и фМЭН-1, а также более 30 микроРНК при сравнении их с группой контроля. МикроРНК, различия в экспрессии которых были выявлены, оказывают регуляторное воздействие, в том числе, на гены, мутации в которых приводят к развитию наследственных аденом гипофиза, а также к клинически схожему фенотипу МЭН-1.

Ранее оценка экспрессии микроРНК в сыворотке крови у пациентов с гМЭН-1 по сравнению с их здоровыми родственниками проводилась в работе Kooblall K.G. и ­соавт. [23]. В их исследовании анализ экспрессии циркулирующих микроРНК производился методом NGS с последующей валидацией методом qRT-PCR, по результатам которого было выявлено, что экспрессия miR-3156-5p значительно снижена у пациентов с гМЭН-1, чем у их родственников. Также в ходе эксперимента на BON-1 клетках НЭО поджелудочной железы с нокаутом гена MEN1 было отмечено, что подавление miR-3156-5p может быть следствием снижения экспрессии менина. В нашей работе различий по данной микроРНК выявлено не было.

Согласно данным литературы, miR-24-1 [24], а также miR-4258, miR-664, miR-1301 [25] и miR-199b-5p [26], определенные в тканях аденом ОЩЖ гМЭН-1 и спорадических ПГПТ методом RT-qPCR, могут являться потенциальными прогностическими и диагностическими биомаркерами. Повышение уровня miR-24, по данным Luzi E. и соавт., коррелирует со снижением уровня менина, что может объяснять второй «удар» инактивации гена MEN1 в МЭН-1-ассоциированных опухолях без потери гетерозиготности [24]. В нашем исследовании miR-24 ­статистически значимо не отличалась между группами фМЭН-1 и гМЭН-1 (р=0,999), а также контрольной группой, в равной степени как и miR-4258 и miR-664, а экспрессия miR-1301 была повышена в группе гМЭН-1 по сравнению с фМЭН-1 (р=0,032) и контролем. Повышение miR-1301 в группе гМЭН-1 могло быть связано с большим количеством карциноидов легкого в этой группе [27], где данная микроРНК может являться промотором канцерогенеза.

В исследовании Luzi E. и соавт. [28] у пациента с гМЭН-1 был определен профиль экспрессии микроРНК в гастриноме, НЭО и нормальной ткани поджелудочной железы. Авторы отобрали 7 микроРНК при помощи NGS с дальнейшей валидацией результатов методом RT-qPCR: miR‐378‐3p, miR‐1468‐5p, miR‐625‐5p, miR‐625‐3p, miR‐215‐5p, miR‐1301‐3p; miR‐212‐5p. Так, экспрессия miR‐212‐5p и miR‐1301‐3p была повышена в НЭО поджелудочной железы и гастриноме по сравнению с нормальной тканью. А экспрессия miR‐378‐3p, miR‐1468‐5p, miR‐625‐5p, miR‐215‐5p была снижена в НЭО и гастриноме по сравнению с контролем. В нашем исследовании в группе гМЭН-1 выявлена повышенная экспрессия miR-215‐5p по сравнению с фМЭН-1 (р=0,05) и контролем. В то же время в исследовании Lutsenko A. и соавт. при сравнении профиля экспрессии микроРНК пациентов со спорадической акромегалией и контролем было выявлено снижение уровней miR-4446-3p и miR-215‐5p в группе акромегалии по сравнению с контролем [29], что может объяснять сниженную экспрессию miR-215‐5p в группе фМЭН-1, учитывая наличие акромегалии у всех пациентов в этой группе. Усиление экспрессии miR-215‐5p может быть связано также со злокачественными новообразованиями, так, например, повышение экспрессии miR-215‐5p выявлено у пациентов с аденокарциномой протоков поджелудочной железы [30], с раком желудка [31], пациентов с остеосаркомой по сравнению с контролем [32] и у пациентов с раком молочной железы по сравнению с контрольной группой [33]. Между тем гиперэкспрессия miR-215‐5p в клеточных линиях колоректального рака, наоборот, приводила к значительному снижению клоногенного потенциала, миграции и инвазивности этих клеток [34].

В исследовании Kim C. и соавт. на тканях НЭО поджелудочной железы miR-30a-3p конкурировала с HuD (РНК-связывающий белок, участвующий в посттранскрипционной регуляции мРНК) за связывание с 3ʹ-НТО мРНК p27 (ингибитор циклин-зависимой киназы 1B, кодируемый геном CDKN1B), которое приводит к подавлению прогрессирования клеточного цикла и роста НЭО. Данные показывают, что HuD может являться основным супрессором роста НЭО поджелудочной железы, а miR-30a-3p взаимодействует с ним в контроле экспрессии p27 [35]. В нашем исследовании экспрессия miR-30a-3p была повышена в группе гМЭН-1 по сравнению с фМЭН-1 и контролем, что может быть связано с большим количеством НЭО ПЖ у пациентов с гМЭН-1 и возможным протективным действием, влияя на мРНК p27.

В исследовании He Z. и соавт. проанализировали, как профиль экспрессии микроРНК различался между СТГ-АГ, ПРЛ-АГ и НАГ, а также нормальной тканью гипофиза при помощи NGS и RT-qPCR. Уровни экспрессий miR-34c-3p, miR-34b-5p, miR-338-5p и miR-375 оказались значительно ниже в группе ПРЛ-АГ по сравнению с контролем. В группе НАГ miR-493-5p и miR-124-3p были значительно подавлены, в то время как экспрессия miR-181b-5p была усилена по сравнению с контролем. В образцах СТГ-АГ экспрессия miR-184 была значительно усилена, тогда как экспрессия miR-124-3p была значительно снижена по сравнению с контролем [36]. В нашем исследовании экспрессия онкосупрессорной miR-375 была снижена при гМЭН-1 по сравнению со здоровым контролем, что коррелирует с результатами исследования He Z. и соавт. В другой работе Müssnich P. и соавт. проанализировали различия в характере экспрессии микроРНК между 12 гонадотропиномами и нормальным гипофизом, выявив методом qRT-PCR две микроРНК с пониженной экспрессией (miR-432 и miR-410) и две с повышенной экспрессией (miR-374b и miR-17). В дальнейшем при сравнении гонадотропином, 12 ПРЛ-АГ и 12 СТГ-АГ было выявлено повышение экспрессии miR-410 в большинстве ПРЛ-АГ и в половине СТГ-АГ, в отличие от гонадотропином [37]. В нашем исследовании различий по miR-432, miR-410, miR-17 выявлено не было (p=0,098). Оценка циркулирующих микроРНК в периферической крови и в крови, оттекающей от гипофиза, проводилась также у пациентов с кортикотропиномой по сравнению с карциноидной опухолью другой локализации, где была выявлена более высокая экспрессия miR-383-3p, miR-4290 и miR-6717-5p и miR-302c-3p в крови, оттекающей от гипофиза, и повышенная экспрессия miR-16-5p и miR-145-5p в периферической крови [ 38; 39].

Mao D. и соавт. проанализировали взаимосвязь miR-944 и длинной нкРНК — ген-хозяин 6 малой ядрышковой РНК (SNHG6, участвует в процессах онкогенеза) в тканях инвазивных АГ. Экспрессия miR-944 была подавлена, а SNHG6 усилена в образцах ткани инвазивных АГ по сравнению с неинвазивными. SNHG6 заметно повышал жизнеспособность, миграцию, инвазию и эпителиально-мезенхимальный переход клеток АГ, тогда как miR-944 имела противоположные эффекты. Кроме того, было подтверждено, что miR-944 может соединяться с 3’-НТО RAB11A (участвует в онкогенезе при АГ, активируя сигнальный путь Wnt/β-катенин) и подавлять его экспрессию. Это исследование подтверждает, что ось SNHG6/miR-994/RAB11A может играть решающую роль в регуляции пролиферации, миграции, инвазии и эпителиально-мезенхимального перехода клеток инвазивных АГ [40]. В нашем исследовании экспрессия онкосупрессорной miR-944 была повышена в группе фМЭН-1 по сравнению с контролем, что также может быть связано с отсутствием инвазивных АГ в этой группе.

В исследовании Valassi E. и соавт. проводилась оценка экспрессии циркулирующих микроРНК сыворотки, предположительно участвующих в метаболизме кости, у пациентов с компенсированной акромегалией по сравнению со здоровым контролем. У пациентов с акромегалией были выявлены сверхэкспрессия miR-103a-3p и miR-191-5p и снижение экспрессии miR-660-5p по сравнению с контролем (р<0,001) [41]. В нашем исследовании экспрессия miR-191-5p также была повышена у пациентов в группе фМЭН-1 по сравнению с контролем.

Ранее описанные изменения в экспрессии выявленных циркулирующих микроРНК в нейроэндокринных опухолях сведены в таблице 8.

**Table:** Таблица 8. Обзор различно экспрессирующихся микроРНК в нейроэндокринных опухолях

Ткань/клетки	микроРНК (экспрессия)	Потенциальная биологическая роль	Метод анализа экспрессии микроРНК	Источник
Сыворотка, (МЭН-1 n=9; контроль n=9) BON-1 клетки	miR-3156-5p (↓)	Роль в онкогенезе ГЭП-НЭО	NGS, qRT-PCR	[23]
ОЩЖ (МЭН-1 n=8; спорадические n=4; нормальная n=1)	miR-24-1 (↑)	Роль в онкогенезе аденом ОЩЖ	Нозерн-блот, qRT-PCR	[24]
ОЩЖ (LOH МЭН-1 n=4; МЭН-1 n=3; спорадические n=2)	miR-4258 (↑ или ↓); miR-664 (↑ или ↓); miR-1301 (↑)	Микрочип, qRT-PCR	[25]
ОЩЖ (спорадические n=28; МЭН-1 n=15; нормальные n=27)	miR-199b-5p (↑ или ↓)	Микрочип, qRT-PCR	[26]
Поджелудочная железа (МЭН-1 гастринома n=1; МЭН-1 НЭО ПЖ n=1; нормальная ПЖ n=1)BON-1 клетки	miR‐378‐3p (↓); miR‐1468‐5p (↓); miR‐625‐5p (↓); miR‐625‐3p (↓); miR‐215‐5p (↓) miR‐1301‐3p (↑); miR‐212‐5p (↑)	Роль в онкогенезе ГЭП-НЭО	NGS, qRT-PCR	[28]
Плазма (акромегалия n= 47 в активной стадии; контроль n=28)	miR-4446-3p (↓); miR-215-5p (↓)	—	NGS, qRT-PCR	[29]
Гипофиз (СТГ-АГ n=13; ПРЛ-АГ n=17; НАГ n=42; контроль n=6)	miR-34c-3p (↓); miR-34b-5p (↓); miR-338-5p (↓); miR-375 (↓); miR-493-5p (↓); miR-124-3p (↓); miR-181b-5p (↑); miR-184 (↑)	Регулирование опухолевой прогрессии	NGS, qRT-PCR	[36]
Гипофиз (Гонадотропиномы n=21; СТГ-АГ n=12; ПРЛ-АГ n=12; контроль n=3)клетки HEK-293	miR-374b (↑); miR-17 (↑); miR-432 (↓); miR-410 (↓)	Развитие опухолей гипофиза	Микрочип, qRT-PCR	[37]
Плазма (БИК n=28; АКТГ-эктопия n=13 в активной стадии; контроль n=11)	miR-16-5p (↑); miR-145-5p (↓); miR-7g-5p (↑)	—	qRT-PCR	[38]
Плазма (БИК n=28; АКТГ-эктопия n=13 — в активной стадии; контроль n=11)	miR-383-3p (↑); miR-4290 (↑); miR-6717-5p (↑); miR-302c-3p (↑); miR-1203 (↓); miR-1229-3p (↓); miR-639 (↓)	—	NGS	[39]
Гипофиз (инвазивные АГ n=30; неинвазивные АГ n=30)Линия клеток гипофиза человека HP75	miR-944 (↓)	Роль в онкогенезе АГ	qRT-PCR	[40]
Сыворотка, (компенсированная акромегалия n=27; контроль n=27)	miR-103a-3p (↑); miR-191-5p (↑); miR-660-5p (↓)	—	qRT-PCR	[41]

Ниже будут рассмотрены некоторые публикации, посвященные выявленным в нашем исследовании микроРНК, при других новообразованиях и заболеваниях. Наиболее отличающейся микроРНК между группами гМЭН-1 и фМЭН-1 в нашем исследовании является miR-3613-5p. Согласно исследованиям Ma J. и соавт. [42], Bai X. и соавт. [43] и Cao R. и соавт. [44], экспрессия miR-3613-5p снижена при раке поджелудочной железы, в особенности при метастазах опухоли, влияя на гены-мишени, участвующие в р53, TGFβ (трансформирующий фактор роста бета) сигнальных путях. В исследовании He T. и соавт. выявлено, что miR-3613-5p является ключевым регулятором положительной обратной связи, лежащей в основе взаимосвязи между сигнальными путями NF-κB/RELA и AKT/MAPK, участвующими в онкогенезе [45]. Также, по данным литературы, экспрессия miR-3613-5p повышена у пациентов со светлоклеточным раком почки [46] и у пациентов с резистентным к доксорубицину раком молочной железы [47]. В нашей работе экспрессия потенциального промотора канцерогенеза miR-3613-5p была повышена в группе гМЭН-1 по сравнению с фМЭН-1, что может быть связано с большим количеством НЭО легких и опухолей других органов.

MiR-30a-3p, обозначенная нами ранее, также показала себя как онкосупрессор в других исследованиях. Так, в исследовании Wang W. и соавт. у 83,6% пациентов с гепатоцеллюлярной аденокарциномой (ГЦК) экспрессия miR-30a-3p была значительно снижена (р<0,0001) в опухолях по сравнению с нормальной тканью. Сверхэкспрессия же miR-30a-3p оказывала ингибирующее действие на пролиферацию клеток, индуцировала апоптоз и увеличивала остановку клеток в S-фазе. В ходе эксперимента было выявлено, что miR-30a-3p регулирует функцию клеток ГЦК посредством механизма, включающего снижение экспрессии виментина и MMP3 (металлопротеиназа-3) и восстановление экспрессии E-кадгерина (мембранный белок, участвует в онкогенезе, кодируется геном CDH1) [48]. В работе Tanigawa K. и соавт. было продемонстрировано, что экспрессия miR-30a-3p ингибирует пролиферацию клеток и вызывает остановку клеточного цикла и апоптоз в двух клеточных линиях мелкоклеточного рака легких [49].

По данным Chen T. и соавт., экспрессия miR-335-5p в плазме крови была снижена у пациентов с трижды негативным раком молочной железы по сравнению со здоровым контролем [50], в то время как повышенная экспрессия miR-335-5p приводила к ингибированию ускользания от иммунного ответа, а полное отсутствие miR-335-5p способствовало росту опухоли. В работах на клетках рака поджелудочной железы [51], адренокортикального рака [52] и колоректального рака [53] пониженная экспрессия miR-335-5p была ассоциирована с более неблагоприятным прогнозом заболевания. И наоборот, повышение экспрессии miR-335-5p в основном приводило к снижению пролиферации и миграции опухолевых клеток [53–55]. МикроРНК miR-32-5p проявила себя как онкосупрессор при аденокарциноме поджелудочной железы [56] и как промотор онкогенеза в клетках плоскоклеточного рака полости рта [57].

В исследовании Makler A. и соавт. были отобраны четыре микроРНК: miR-93-5p, miR-339-3p, miR-425-5p и miR-425-3p, различно экспрессирующиеся в плазме крови пациентов с аденокарциномой протоков поджелудочной железы по сравнению с контролем, с целью создания панели для диагностики рака протоков поджелудочной железы. Панель из этих четырех микроРНК имела площадь под кривой 0,885 с чувствительностью 80% и специфичностью 94,7%, что сопоставимо со стандартной маркерной диагностикой CA19-9 [58]. Также повышенная экспрессия miR-425-3p, наряду с miR-584-5p, была обнаружена в плазме крови пациентов с колоректальным раком, и в целом эта микроРНК связана с неблагоприятным прогнозом при раках различных этиологий [59]. В то же время экспрессия miR-141-3p, miR-760, miR-501-3p также была различно изменена при колоректальном раке [60–62], раке поджелудочной железы [63][64] и раке легкого [65–67]. Повышение экспрессии miR-141-3p способствовало пролиферации, миграции и инвазии клеток опухоли, в то время как miR-501-3p проявляла себя как потенциальный онкоген при колоректальном раке и раке протоков ПЖ и как онкосупрессор при мелкоклеточном раке легких. MiR-760 проявляла себя как онкосупрессор. Изменения экспрессии микроРНК miR-1976, miR-144-5p, miR-532-3p, miR-98-5p также в большинстве своем связаны со злокачественными новообразованиями, такими как рак яичника [68][69], рак легкого [70–73], колоректальный рак [74–77], рак поджелудочной железы [78][79].

МикроРНК miR-576-5p играет важную роль в развитии различных видов рака человека. Так, уровень экспрессии miR-576-5p в тканях папиллярного рака щитовидной железы (ПРЩЖ) и клетках ПРЩЖ (TPC-1) был значительно повышен, что способствует пролиферации, миграции и инвазии клеток TPC-1, а также индукции активации сигнального пути Akt/PKB (участвует в онкогенезе) [80]. В тканях аденокарциномы толстого кишечника была также выявлена усиленная экспрессия miR-576-5p, которая заметно способствовала пролиферации, миграции и инвазии клеток через регулятор роста нейронов 1 (NEGR1), который может являться основной терапевтической мишенью при аденокарциноме толстого кишечника [81]. Также повышенная экспрессия miR-576-5p была выявлена при раке эндометрия [82], где miR-576-5p способствовала пролиферации и метастазированию клеток рака эндометрия путем ингибирования экспрессии ZBTB4.

Все вышеуказанные микроРНК (табл. 9) были повышены в группе гМЭН-1 по сравнению с фМЭН-1 и контролем, что может быть, скорее всего, следствием протективного действия этих микроРНК в группе гМЭН-1 и большого количества злокачественных новообразований у этой группы пациентов.

**Table:** Таблица 9. Обзор полученных в рамках исследования микроРНК при других образованиях Примечание: Cr — карцинома; ПЖ — поджелудочная железа; МЖ — молочная железа; ГЦК — гепатоцеллюлярная карцинома; ПРЩЖ — папиллярный рак щитовидной железы; (↓) — сниженная экспрессия; (↑) — повышенная экспрессия.

МикроРНК	Ткань/клетки (экспрессия)	Потенциальная биологическая роль	Источник
miR-3613-5p	Cr ПЖ (↓)	Промотор канцерогенеза	[42] [43] [44]
Cr легкого (↑)	[45]
Светлоклеточный Cr почки (↑)	[46]
Cr МЖ (↑)	[47]
miR-30a-3p	ГЦК (↓)	Онкосупрессор	[48]
Мелкоклеточный Cr легких (↓)	[49]
miR-335-5p	Трижды негативный Cr МЖ (↓)	Онкосупрессор	[50]
Cr ПЖ (↓)	[51]
АКР (↓)	[52]
Колоректальный Cr (↓)	[53]
Cr МЖ (↓)	[54]
Cr мочевого пузыря (↓)	[55]
miR-32-5p	Cr ПЖ (↓)	Онкосупрессор	[56]
Плоскоклеточный Cr полости рта (↑)	Промотор канцерогенеза	[57]
miR-425-3p	Cr протоков ПЖ (↑)	Промотор канцерогенеза	[58]
Колоректальный Cr (↑)	[59]
miR-141-3p	Колоректальный Cr (↑)	Промотор канцерогенеза	[60]
Cr легкого	[65]
miR-501-3p	Колоректальный Cr (↑)	Промотор канцерогенеза	[62]
Cr протоков ПЖ (↑)	[64]
Мелкоклеточный Cr легких (↓)	Онкосупрессор	[67]
miR-760	Колоректальный Cr (↓)	Онкосупрессор	[61]
Cr ПЖ (↓)	[63]
Немелкоклеточный Cr легких (↓)	[66]
miR-1976	Немелкоклеточный Cr легких (↓)	Онкосупрессор	[71]
Колоректальный Cr (↓)	[77]
Cr ПЖ и печени (↑)	Промотор канцерогенеза	[78]
miR-144-5p	Немелкоклеточный Cr легких (↓)	Онкосупрессор	[72]
Колоректальный Cr (↓)	[74]
miR-532-3p	Cr яичников	Онкосупрессор	[68]
Немелкоклеточный Cr легких (↓)	[70]
Колоректальный Cr (↓)	[76]
Cr ПЖ (↓)	[79]
miR-98-5p	Cr яичников	Промотор канцерогенеза	[69]
Немелкоклеточный Cr легких (↓)	Онкосупрессор	[73]
Колоректальный Cr	[75]
miR-576-5p	ПРЩЖ (↑)	Промотор канцерогенеза	[80]
Cr толстого кишечника (↑)	[81]
Cr эндометрия (↑)	[82]

В целом при сравнении гМЭН-1 и фМЭН-1 не выявлено различий в экспрессии miR-24, что могло бы натолкнуть на мысль о схожей посттранскрипционной регуляции и вовлеченности сигнальных путей, в которых участвует менин, однако различий по данной микроРНК не было выявлено и при сравнении обеих групп с контролем. Также отмечено количество одинаково различающихся микроРНК между группами гМЭН-1/фМЭН-1 и гМЭН-1/контролем (miR-25-5p, miR-576-5p, miR-532-3p, miR-30a-3p, miR-215-5p). Таким образом, пилотная работа показала различно экспрессирующиеся микроРНК в периферической крови, измеренные методом NGS, которые сильно отличаются у пациентов с мутацией в гене MEN1, но в меньшей степени в ­группах ­фенокопий и ­контроля, что, по всей видимости, отражает влияние именно мутации в гене на общий пул ­микроРНК.

## Ограничения исследования

Ограничениями данного исследования являются малый размер выборки, различия по возрасту (использован age-adjustment) и аденомам гипофиза с разным типом секреции в группах гМЭН-1 и фМЭН-1. В нашем исследовании также проводилась оценка микроРНК из периферической крови, и мы, скорее всего, не можем исключить, что некоторые изменения могут отражать специфические действия в других тканях. Однако, учитывая воздействие указанных патологий на весь организм, можно предположить, что полученные из периферической крови микроРНК, вероятно, могут отражать изменения, вызванные именно этими заболеваниями. По результатам представленного исследования установлена гипотеза о различиях в экспрессии циркулирующих микроРНК между представленными группами. Для подтверждения выявленных результатов необходимо провести валидизацию, расширив выборку пациентов с использованием метода RT-qPCR, а также включив пациентов с другим типом секреции АГ в группу фенокопий.

## ЗАКЛЮЧЕНИЕ

Впервые в нашей работе изучены различия в экспрессии циркулирующих микроРНК плазмы крови у пациентов с генетически подтвержденным синдромом МЭН-1, его фенокопиями и контрольной группой методом высокопроизводительного секвенирования. Отобраны наиболее отличающиеся по экспрессии микроРНК среди групп для дальнейшей валидации результатов методом RT-qPCR (в группах генетически подтвержденного МЭН-1 против фенокопий синдрома — miR-3613-5p, miR-335-5p, miR-32-5p, miR-425-3p, miR-25-5p, miR-576-5p, miR-215-5p, miR-30a-3p, miR-141-3p, miR-760, miR-501-3p; против контроля — miR-1976, miR-144-5p miR-532-3p, miR-375; а также в группах фенокопий синдрома МЭН-1 против контроля — miR-944, miR-191-5p, miR-98-5p). В целом полученные результаты, скорее всего, могут отражать взаимосвязь выявленных микроРНК и их генов-мишеней на транскрипционные факторы, молекулы сигнальных путей, клеточные циклы, дифференцировку клеток и гены-онкосупрессоры.

## ДОПОЛНИТЕЛЬНАЯ ИНФОРМАЦИЯ

Источники финансирования. Исследование выполнено в рамках гранта Российского научного фонда №19-15-00398.

Конфликт интересов. Авторы декларируют отсутствие явных и потенциальных конфликтов интересов, связанных с содержанием настоящей статьи.

Участие авторов. Все авторы одобрили финальную версию статьи перед публикацией, выразили согласие нести ответственность за все аспекты работы, подразумевающую надлежащее изучение и решение вопросов, связанных с точностью или добросовестностью любой части работы.
